# Beyond animal models: using human pluripotent stem cell-derived stem and progenitor cells to predict cytotoxicity in normal tissues

**DOI:** 10.3389/pore.2026.1612490

**Published:** 2026-08-20

**Authors:** Katalin Vincze, Nóra Varga, Szilárd Tóth, Anna Kamilla Kis, Rita Béres, János Miklós Réthelyi, Zoltán János Veréb, Zsuzsa Erdei, Balázs Sarkadi, Ágota Apáti

**Affiliations:** 1 Institute of Molecular Life Sciences, HUN-REN Research Center for Natural Sciences, Budapest, Hungary; 2 Department of Psychiatry and Psychotherapy, Semmelweis University, Budapest, Hungary; 3 Salus Ltd., Budapest, Hungary; 4 Regenerative Medicine and Cellular Pharmacology Laboratory, Department of Dermatology and Allergology, University of Szeged, Szeged, Hungary; 5 Innocell Ltd., Budapest, Hungary

**Keywords:** cytotoxicity, induced pluripotent stem cells (iPSCs), isogenic *in vitro* model, lineage-specific toxicity, mesenchymal stem cells

## Abstract

**Background:**

The clinical efficacy of anticancer drugs is often limited by dose-dependent toxicity to normal cells, particularly to rapidly dividing stem and progenitor cell populations. Traditional preclinical toxicity screening relies on animal models or primary human cells/immortalized lines, both of which have significant limitations regarding scalability, reproducibility, genetic diversity, and translational relevance.

**Objective:**

This study aims to establish and validate a human pluripotent stem cell -based platform for profiling the toxicity of cytostatic drugs on healthy, dividing cell populations, and to compare their sensitivity to that of cancer cell lines.

**Methods:**

Two hiPSC lines (male and female) were reprogrammed from umbilical cord blood cells, fully characterized, and differentiated into neural progenitors and mesenchymal derivatives. Dose–response curves were generated using a dilution series of four anticancer drugs and four other pharmacologically active compounds based on cell viability. Toxic half-maximal values (IC_50_) were determined for each cell type and compared to those obtained from conventional cancer cell lines.

**Results:**

The highest sensitivity to DNA-damaging agents was exhibited by pluripotent stem cells, followed by neural progenitors, with mesenchymal derivatives being the least sensitive. Notably, except for mesenchymal cells, both pluripotent stem cells and their differentiated derivatives were more sensitive to cytostatics than cancer cell lines.

**Conclusion:**

Human iPSC-derived stem/progenitor cells recapitulate the *in vivo* sensitivity of healthy dividing cell populations to chemotherapy. This platform offers unlimited, genetically consistent, and reproducible access to human cells, rendering it as a powerful complementary approach or even an alternative to animal models and primary human cells for early-phase drug safety profiling.

## Introduction

A major limitation of conventional anticancer chemotherapy is its lack of selectivity for malignant tissues, leading to severe damage to rapidly renewing healthy tissues. Consequently, clinical dose-limiting toxicities—such as myelosuppression, mucositis, hair loss, infertility, and gastrointestinal damage—result from the destruction of healthy stem and progenitor cell compartments, including hematopoietic, intestinal crypt, and germ cell populations [1–3]. The inability to distinguish between malignant and healthy proliferating cells directly narrows the therapeutic window and treatment options. Rapid and accurate preclinical prediction of toxicity in normal human stem and progenitor populations is an urgent, unmet need [4].

This problem is exacerbated by the limited predictive value of traditional preclinical models, as more than 90% of compounds that demonstrate safety and efficacy in animal studies ultimately fail in human clinical trials due to unexpected toxicity or lack of efficacy [5]. In order to improve human relevance and adhere to the 3Rs (replace, reduce, refine animal models), global regulatory frameworks have undergone a significant paradigm shift towards new approach methodologies (NAMs). Landmark initiatives—including the US FDA (Food and Drug Administration) Modernization Act 2.0 [6], the subsequent FDA Strategic Roadmap 2025 (FDA, 2025), the revised guidelines of the European Medicines Agency (EMA) and the 3R Working Group (EMA, 2023; EMA 3R Working Group Biennial Report, 2023–2024), and international harmonization efforts (IMRWG3R, 2024)—now formally recognize and encourage human cell-based studies to establish drug safety and efficacy.

Despite the above regulatory directions, the preclinical application of healthy human stem and progenitor controls remains rare, and their lack limits more accurate *in vitro* toxicity assessments. When using healthy human controls (primary cells or cell lines) to calculate the selectivity index (SI) of a compound [7, 8], current experimental setups have significant limitations:

- Inadequately representative models: Many studies rely on a single normal human control line, such as fibroblasts or epithelial cells. This does not reflect clinical reality, as the primary off-target effects of chemotherapy are related to actively dividing stem/progenitor cells, not quiescent fibroblasts.
- Diverse genetic background: Although the inclusion of multiple normal human cell types may improve the estimation of compound selectivity [9], these lines typically originate from different donors. This genetic heterogeneity makes it difficult to determine whether changes in drug sensitivity arise from true tissue-specific vulnerabilities or from underlying donor variability.
- *In vitro* culture limitations: The procurement of primary human cells is limited by finite proliferative capacity and rapid functional decline *ex vivo* [10–12]. Immortalized cell lines offer better reproducibility, but often harbor genetic mutations and altered cell cycle regulation, or are themselves of tumor origin, making their toxicological data difficult to translate into physiological human responses [13, 14].

Human induced pluripotent stem cells (hiPSCs) offer an ethical, non-malignant alternative to embryonic stem cells and tumor lines, providing a virtually inexhaustible source of human cells for disease modeling and drug screening [15–17]. Because undifferentiated hiPSCs exhibit a rapid cell cycle, stringent DNA damage checkpoints, and a robust p53-mediated apoptotic response [18, 19], their heightened sensitivity to genotoxic stress effectively models the vulnerability of rapidly dividing healthy tissue [20]. Crucially, a single hiPSC line can be differentiated into multiple lineage-specific cell types. This allows for direct comparison of intrinsic, lineage-dependent toxicity profiles while minimizing the confounding effects of inter-donor genetic heterogeneity [4, 21].

The choice of somatic donor tissue directly impacts the stability and quality of the resulting hiPSC lines. Umbilical cord blood-derived cells are biologically young, exhibiting high telomerase activity [22], fewer somatic mutations [16, 23], and a “naïve” epigenetic state that facilitates high reprogramming efficiency [24]. Collected via non-invasive, routine biobanking methods [25], these newborn cells possess minimal immunological memory and lack the age-related cellular damage, chronic inflammation, and epigenetic drift typical of adult donor cells—alterations that can persist post-reprogramming [26–28]. While their early developmental origin may result in altered metabolic profiles or immature drug-metabolizing enzyme activity relative to adult cells [29], their high baseline fidelity makes them an ideal starting material for predictive toxicological platforms.

Despite clinical evidence showing that women face a higher risk of adverse drug reactions (ADRs) [30], biological sex remains severely underrepresented in preclinical research [31, 32]. This omission is critical because sex-dependent differences in drug sensitivity and mitochondrial stress responses manifest at the cellular level [33]. To align with evolving regulatory mandates emphasizing sex as a biological variable, this study utilizes two established cord blood-derived hiPSC lines: KUT-2 (XX) and KUT-6 (XY), enabling the systematic evaluation of sex-specific cytotoxic liabilities.

One of the key advantages of hiPSC-based systems is the ability to generate and study multiple cell types derived from the same genetic background. This enables the investigation of lineage-specific toxicological responses, as well as the underlying mechanism-specific sensitivities [4]. In the present study, we hypothesized that different cell types may exhibit distinct sensitivities to cytotoxic compounds. To evaluate cell type-specific cytotoxicity across a biological gradient [21], we examined undifferentiated hiPSCs alongside their differentiated derivatives representing distinct embryonic germ layers. hiPSCs are already routinely and successfully used in embryotoxicity and teratogenicity assays [34, 35]. Their differentiated stem/progenitor derivatives, although often retaining a fetal-like phenotype, provide valuable *in vitro* models for tissue-specific stem cell populations and enable the investigation of lineage-dependent toxic responses. The nervous system is particularly sensitive to toxicants, so neural models are essential for studying neurotoxicity [36]. Neurogenesis produces Neural Stem Cells (NSCs), which can self-renew and generate other types of neural tissue cells including neurons, astrocytes and oligodendrocytes [37]. hiPSC-derived NSCs represent an ectodermal model that is highly sensitive to metabolic stress, ion channel disruption and proteostasis impairment [36] and can be used as a model of the developing brain. Our other neural model is based on a specific differentiation protocol to generate hippocampal Neural Progenitor Cells (hNPCs) from hiPSCs. Since the adult dentate gyrus of the hippocampus contains neuronal stem cells that give rise to immature and subsequently mature functional granule neurons [38], hNPCs can be used as a model not only of the developing hippocampus, but also of adult neurogenesis [39]. In contrast, Mesenchymal Stem Cell-like cells (MSCs), differentiated from hiPSCs, are mesodermal stromal models. MSCs play a key role in the stromal microenvironment, contribute to tissue regeneration, and are characterized by lower proliferative activity, a different metabolism, and a more resistant stress response profile [40–42]. In addition to modeling stromal cells involved in regenerative processes, they may also contribute to the understanding of tumor-associated mesoderm cells that appear in the tumor environment [43]. To characterize cytotoxic responses across mechanistically distinct cellular stress pathways, we assembled a panel of eight pharmacologically active compounds with diverse and well-defined modes of action (Supplementary Table 1). This approach enables the identification of pathway-specific vulnerabilities while allowing comparison of cell type–specific sensitivities across hiPSCs, MSCs, and NPCs [4, 21].

The selected compounds in this study can be broadly grouped into two functional categories. The first comprises agents that primarily target DNA integrity and replication. Doxorubicin exerts its cytotoxic effects through multiple mechanisms, including DNA intercalation, topoisomerase II inhibition, and free radical-mediated DNA-damage, ultimately inducing, apoptosis, autophagy, senescence, necrosis, and metabolic alterations. Due to its high cytotoxic efficacy, it is widely used in the treatment of both adult and pediatric hematological malignancies and solid tumors, (breast, liver, bile ducts, endometrial tissue, osteosarcomas, esophagus) [44, 45]. Unfortunately, normal cells are also sensitive to doxorubicin, which primarily affects the heart, brain, liver, and kidneys, often long after the treatment. This cytotoxic profile underlines the importance of a precise application dose [45]. Mitoxantrone acts as DNA intercalators and topoisomerase II inhibitors, promoting strand breaks and replication arrest [46]; SN-38 stabilizes the topoisomerase I–DNA cleavage complex, inducing replication-associated single- and double-strand breaks [47]; and Laromustine functions as a sulfonylhydrazine alkylating agent that induces O6-guanine DNA alkylation, leading to the formation of DNA crosslinks, while simultaneously inhibiting the repair enzyme O6-alkylguanine DNA transferase [48]. The second category encompasses compounds that modulate non-genotoxic stress pathways: Deferiprone exerts its effects by chelating intracellular iron, thereby disrupting iron-dependent mitochondrial processes and promoting the accumulation of reactive oxygen species (ROS), including mitochondrial superoxide [49]; Celecoxib, a selective COX-2 inhibitor, exerts pro-apoptotic and anti-proliferative effects through inhibition of COX-2–mediated Prostaglandin E2 (PGE2) signaling and its downstream pathways, including NF-κB and Akt [50]; Phenothiazine interferes with multiple tumor-promoting pathways, including PDK1/Akt, MAPK/ERK1/2, and Akt/mTOR, and can promote lysosome-mediated cell death while inhibiting drug efflux pumps [51], while Quinacrine is primarily an antimalarial agent, but it is also known to have antitumor effects; it is capable of inhibiting phospholipase A and intercalating into DNA [52, 53]. Together, this panel enables a systematic evaluation of how hiPSC-derived cell types of distinct lineages respond to genotoxic, oxidative, autophagic, and signaling-mediated cytotoxic stimuli, thereby providing mechanistic insight into lineage-dependent differences in cellular stress tolerance.

Taken together, the present study employs a multidimensional experimental framework that integrates several key design principles of modern preclinical research. By utilizing hiPSC lines derived from umbilical cord blood of both a male and a female donor, this study combines the biological advantages of neonatal-derived cells with the ability to explore sex as a biological variable within a controlled genetic context. Differentiation of these lines into mesenchymal stem cells and neural progenitor cells, representing distinct germ layer derivatives, enables the investigation of lineage-specific cytotoxic responses. The application of a mechanistically diverse compound panel further allows the systematic interrogation of multiple cytotoxic pathways across cell types. This approach is designed to generate translatable, human-relevant data that addresses recognized limitations of conventional preclinical models, while contributing to the emerging evidence base for hiPSC-based platforms as physiologically meaningful tools for drug safety assessment.

## Materials and methods

Detailed information about the materials used can be found in Supplementary Table 2.

### Generation of hiPSC lines from umbilical cord blood

According to the permission for reprogramming and the study by the Human Reproduction Committee of the Hungarian Health Science Council (ETT HRB- (Approval n. 3883-2/2018/EÜIG), umbilical cord blood samples were obtained after written informed consent. Umbilical cord blood mononuclear cells (UCB-MNCs) were isolated via BD Vacutainer CPT tubes (sodium citrate) per manufacturer protocols. UCB-MNCs were seeded at 10^5^ cells/well in 24-well plates using supplemented StemPro-34 medium containing 2 mM GlutaMax, 10 µM β-mercaptoethanol, 1% Antibiotic-Antimycotic and cytokines: 20 ng/mL IL-3, 100–100 ng/mL IL-6, FLT-3L and SCF. Following 2 days of expansion with daily half-medium changes, cells were transduced using the CytoTune-iPS 2.0 Sendai Reprogramming Kit (Oct3/4, Sox2, Klf4, c-Myc). Vectors were removed after 24 h, and cells were maintained in UCB-MNC medium for an additional 48 h. Transduced cells were subsequently transitioned onto mitomycin C-treated CF-1 feeder cells in cytokine-free StemPro-34 medium. After 2 days, the culture was transitioned to hiPSC medium (KO-DMEM, 10% KO-Serum Replacement, 100 mM NEAA, 2 mM GlutaMax, 10 mM β-mercaptoethanol, 4 ng/mL bFGF, 1% Antibiotic-Antimycotic) via gradual replacement. Emerging colonies were manually picked and expanded on feeder before being transitioned to feeder-free conditions on hESC-qualified Matrigel-coated plates in mTeSR1 medium. Two hiPSC lines were established, characterized, and registered at hpscreg.eu under the following names: RCNSi009-A for KUT-2 and RCNSi0010-A for KUT-6.

### iPSC cell culture

KUT-2 and KUT-6 hiPSC lines were cultured on plates coated with Matrigel and maintained in mTeSR1 medium. Culture medium was refreshed daily. Cells were passaged every 3–4 days at an approximate ratio of 1:10 using ReLeSR, and subsequently replated onto freshly coated Matrigel surfaces in mTeSR1 medium.

### Spontaneous differentiation of hiPSC lines

To demonstrate pluripotency, we examined the differentiation of the three germ layer directions during spontaneous differentiation. The spontaneous *in vitro* differentiation potential was examined by the formation of embryoid bodies (EBs), as described previously [54]. The EBs were cultured for 6 days in a free-floating culture and then maintained for 6 and 12 days on gelatin-coated Nunc Lab-Tek confocal chambers in DMEM medium supplemented with 10% ES tested FBS and 2 mM GlutaMAX™.

### Directed differentiation of hiPSCs

The generation of hippocampal neuronal progenitor cells has been described previously [55]. The hNPCs were maintained on Matrigel coated plates in DMEM/F-12, 2 mM GlutaMAX™/N2/B27 medium containing 1% FGF2, 2% laminin and 1% Antibiotic-Antimycotic. For neural stem cell (NSC) induction, PSC Neural Induction was used according to the manufacturer’s instructions. The NSCs were cultured in Neuronal Expansion medium (AdvancedTM DMEM⁄F-12 and Neurobasal® Medium in 1:1 ratio, 2% Neuronal Induction Supplement, 1% Antibiotic-Antimycotic on Matrigel coated plates. We performed immunocytochemical staining to identify neural progenitor markers.

Mesenchymal-like cells (MSC) were generated from hiPSC lines as described previously [56]. MSCs were cultured in DMEM medium supplemented with 10% ES tested FBS and 2 mM GlutaMAX™. For adipogenic and osteogenic differentiation, Human Mesenchymal Stem Cell Functional Identification Kit was used according to the manufacturers’ instructions. To determine MSC markers, flow cytometry was used.

### Human tumor cell line culturing

The human uterine sarcoma cell line Mes-Sa was obtained from American Type Culture Collection (ATCC). The human ovarian serous adenocarcinoma cell line OVCAR-8 (OVC-8) was provided by the National Cancer Institute Developmental Therapeutics Program of the National Institute of Health (NIH NCI DTP) as part of the NCI-60 human tumor cell line panel. Mes-Sa cells were cultured in DMEM, while OVC-8 cells were maintained in RPMI, both supplemented with 10% FBS, 100 U/mL penicillin, 100 μg/mL streptomycin, and 2 mM L-glutamine.

### Karyotype and STR analysis

The genetic identity and chromosomal integrity of the hiPSC lines were verified by short tandem repeat (STR-data can be presented upon request) profiling and G-banding karyotype analysis. These analyses were performed by UD-GenoMed Medical Genomic Technologies Ltd.

### Flow cytometry measurements

Flow cytometric analysis of SSEA-4 and MSC marker expression was carried out as described previously [54], with minor modifications. Briefly, single-cell suspensions were generated using StemPro Accutase. Cells were then incubated with PE-conjugated anti-human SSEA-4 antibody (1:100) in PBS containing 0.5% bovine serum albumin at 37 °C for 30 min. To exclude non-viable cells, propidium iodide staining was applied during analysis. Appropriate isotype controls were included in all experiments (1:100). MSC identity was assessed by flow cytometry using antibodies against CD73, CD44, CD45, CD31, CD34, and CD90. Appropriate isotype controls were included in all experiments.

### Immunocytochemistry

For immunocytochemical analysis, cells were seeded onto eight-well Nunc Lab-Tek II chambered cover glass slides. Cells were fixed and permeabilized using 4% paraformaldehyde (PFA) in DPBS for 15 min at room temperature. Following fixation, samples were blocked for 60 min at room temperature in a blocking buffer consisting of DPBS supplemented with 2 mg/mL BSA, 0.1% Triton X-100, and 1% fish gelatin, with or without 5% goat serum. Cells were subsequently incubated for 60 min at room temperature with primary antibodies diluted in blocking buffer. Pluripotency markers included OCT3/4 (1:50) and NANOG (1:100), while lineage-specific markers included AFP (α-fetoprotein) (1:500), SMA (α-smooth muscle actin) (1:500), and β-III tubulin (1:2000). Neuronal progenitor markers included Sox1, (1:100) Sox2 (1:20), Pax6 (1:100), and Nestin (1:250). After washing with DPBS, cells were incubated for 60 min at room temperature with Alexa Fluor 647-conjugated goat anti-mouse and Alexa Fluor 594-conjugated donkey anti-goat secondary antibodies (1:250). Nuclear staining was performed using DAPI. Samples were imaged using a Zeiss LSM 710 confocal laser scanning microscope.

### RNA isolation and gene expression analysis

Total RNA was isolated from KUT-2 and KUT-6 cells and differentiated derivatives using TRIzol reagent. RNA integrity was assessed by agarose gel electrophoresis, while concentration and purity were determined using a Nanodrop spectrophotometer. For mRNA expression analysis, cDNA was synthesized from 400 ng total RNA using the Promega Reverse Transcription system according to the manufacturer’s protocol. Expression levels of SEV, BRY, OCT4, GATA4, NANOG, AFP, and Nestin were quantified using TaqMan® gene expression assays. Quantitative PCR reactions were performed on a StepOne™ Real-Time PCR System (Applied Biosystems) following the manufacturer’s recommendations. Gene expression levels were normalized to endogenous control RPLP0.

### Cytotoxicity measurements

After trypsinization, both cancer cell lines and the MSCs were seeded on 384-well microplates in a density of 2500 cells/well in 40 μL complete medium. Prior to seeding hiPSC and NSC lines, 20 μL of cold Matrigel was dispensed into pre-chilled 384-well plates; for hNPC cultures, cold Matrigel was used instead. Plates were incubated at 37 °C for 1 h, after which excess coating solution was removed manually using a vacuum hand operator (Vacuboy, Integra Biosciences), and 2500 cells/well in 40 μL cell seeding was performed immediately, as described for the other lines. The following day, when cells were attached, serial dilution of the test drugs were aspirated in an additional 20 μL complete medium, and plates were incubated for 72 h.

Cytotoxicity was assessed by adding resazurin-based PrestoBlue viability reagent to a final concentration of 10%, followed by 1 h incubation, after which fluorescence of the reduced dye (resorufin) was measured at 555/585 nm ex./em. wavelengths with an EnSpire (Perkin Elmer) plate reader. Cytotoxicity testing-related liquid aspirations were performed by a Hamilton StarLet robot.

Raw data files were uploaded into our custom program, which performed automated data normalization, sigmoidal curve fitting, and pIC_50_ calculation [57]. The mean and standard deviation of pIC_50_ values were determined from at least three independent experiments. IC_50_ values were then obtained by logarithmic transformation, with corresponding ± SD reported, noting that symmetric SD values in pIC_50_ become asymmetric after transformation.

### Statistical analysis

Statistical analyses were performed using GraphPad Prism. Differences in cytotoxicity among cell types and compounds were evaluated using ordinary two-way ANOVA with Tukey’s multiple comparisons *post hoc* test. The significance threshold (α) was set at 0.05. Cell identity was used as the row factor, while compound identity was used as the column factor. Type III sum-of-squares analysis was applied to determine the contribution of each factor and their interaction to the total variance (Supplementary Tables 3A,B). Adjusted p values from Tukey’s multiple comparisons test were used to compare estimated marginal means between cell types. Pairwise comparisons were performed among all cell derivatives across the KUT-2 and KUT-6 backgrounds to identify statistically significant differences in cytotoxicity. Tukey’s correction was applied to reduce the probability of type I error arising from multiple comparisons, and adjusted p values < 0.05 were considered statistically significant.

## Results

Our aim was to determine whether pluripotent and lineage-committed derivatives of human umbilical cord blood-derived induced pluripotent stem cells (hiPSCs) exhibit differential sensitivity to cytotoxic compounds. We hypothesized that pluripotent hiPSCs, due to their rapid proliferation rate and increased susceptibility to DNA damage, would display higher sensitivity, whereas differentiated mesenchymal stem cells (MSCs) and neural progenitor cells (NPCs) would show distinct, lineage-specific stress responses resulting in different patterns of drug sensitivity. Two independent hiPSC lines derived from different donors with sex differences were used in this study, allowing limited assessment of donor-dependent variability in drug responses at the pluripotent stage. Since our goal here was to provide detailed examples for NAMs, potentially replacing some of the animal studies, we had to comply with the regulatory requirements (see ref 6). These include defined test methodologies; establishing the relevance within the context of use; and demonstration of reliability and robustness. Thus, a detailed characterization of the applied iPS cells and their differentiated derivatives, as well as their response to drug treatment had to be performed.

### Generation and validation of hiPSC lines derived from umbilical cord blood

**FIGURE 1:**
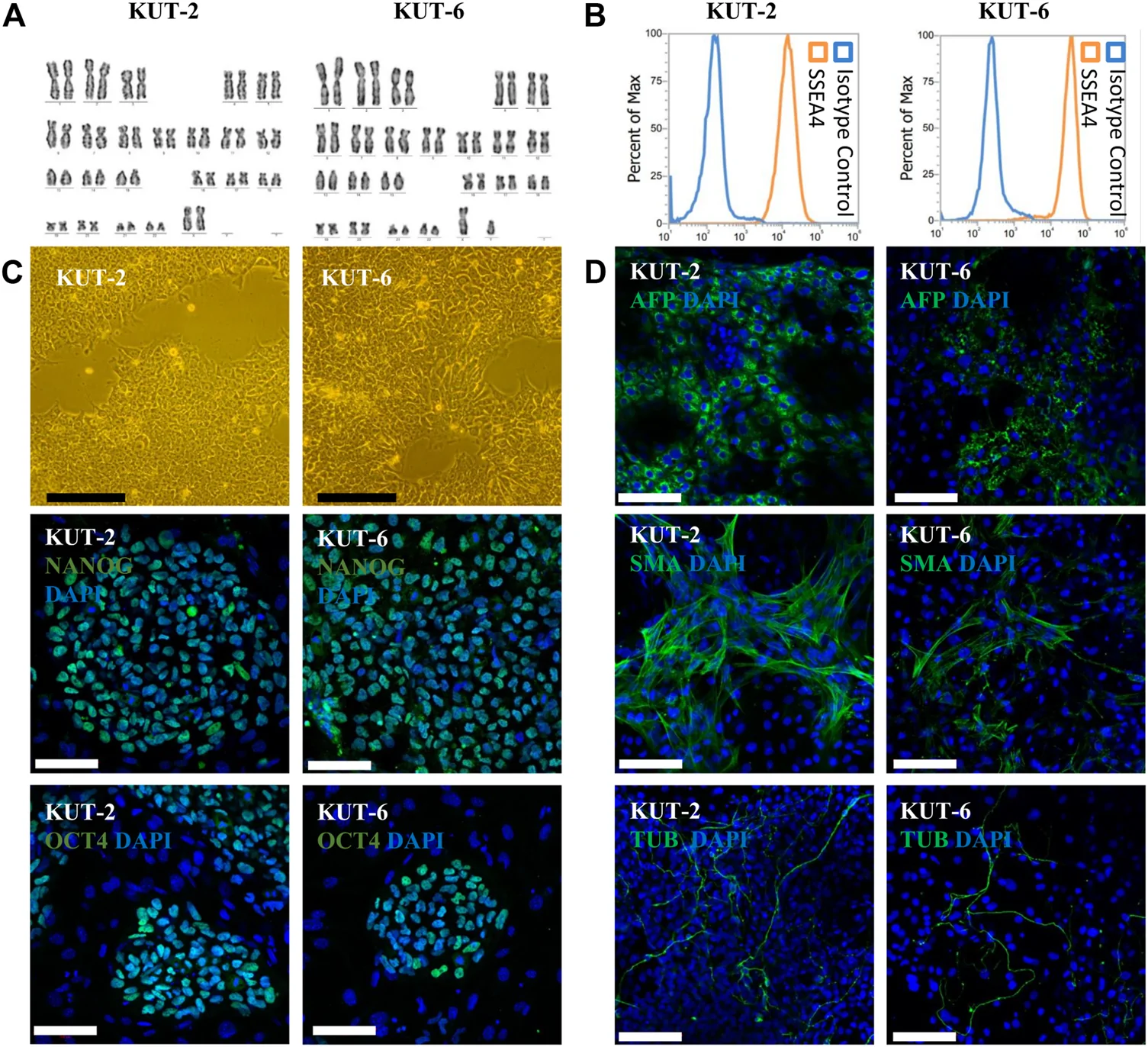
Characterization of induced pluripotent stem cell lines. **(A)** Karyotype analysis of hiPSC clones. **(B)** Cell surface expression of SSEA4 by flow cytometry (98%). **(C)** Morphology and immunofluorescence staining of pluripotency markers (NANOG and OCT4). **(D)** Immunofluorescence staining of germ layer markers (AFP as endoderm, SMA as mesoderm, and β-III tubulin (TUB) as ectoderm markers). Scale bar size; black: 200 μM; white: 100 µM.

Two distinct hiPSC lines (KUT-2- female and KUT-6 male) were generated from umbilical cord blood–derived mononuclear cells using Sendai virus–mediated reprogramming. Sendai virus reprogramming is widespread because the reprogramming factors (OCT4, SOX2, cMYC and KLF4) they are expressed only transiently and in the cytoplasm, do not integrate into the nucleus, and are diluted with cell division. Thus, reprogramming occurs without a genetic “trace”. The clearance of the virus should be checked after 10 passages. Clonal lines exhibiting normal diploid karyotypes were selected for further analysis, confirming genomic integrity (Figure 1A).

In order for hiPSC-based models to produce reproducible results, the starting hiPSCs must be thoroughly characterized according to the strict standards of the field. The most commonly used characterization methods include assessment of morphology, immunostaining for surface (SSEA4) and nuclear pluripotency markers (OCT4, NANOG) and verification of the pluripotent state by spontaneous differentiation. During spontaneous differentiation induced by serum-containing three dimensional culture conditions, pluripotent cells give rise to cell types representing all three germ layers. Ectodermal, mesodermal and endodermal cell populations appearing after 12 days of differentiation must be detected using at least two independent methods (e.g., immunostaining and RT-PCR) and the loss of pluripotency markers must also be confirmed.

Both hiPSC lines displayed typical human pluripotent stem cell morphology, characterized by compact colonies with defined borders and a high nuclear-to-cytoplasmic ratio. Pluripotency was further confirmed by immunofluorescence staining, demonstrating robust expression of the core transcription factors OCT4 and NANOG (Figure 1C).

Flow cytometric analysis revealed high surface expression of SSEA4 (approximately 96%–98%), indicating a homogeneous pluripotent population (Figure 1B).

The differentiation capacity of the hiPSC lines was assessed through spontaneous embryoid body (EB) formation. Following differentiation, cells lost pluripotency markers (OCT4 and NANOG see Supplementary Figure 1) and expressed lineage-specific markers representing all three germ layers, including AFP (endoderm), SMA (mesoderm), and β-III tubulin (ectoderm), as confirmed by immunofluorescence staining (Figure 1D). The differentiation capacity to the three germ layers was also validated by qPCR (Supplementary Figure 1).

### Quality control and genomic stability of hiPSC lines

Comprehensive quality control analyses confirmed the stability and suitability of the generated hiPSC lines for downstream applications. Clearance of the Sendai viral vectors was verified by RT-PCR, demonstrating the absence of residual reprogramming vectors after hiPSC establishment (Supplementary Figure 1A). Karyotype analysis confirmed normal chromosomal composition, while STR profiling verified cell line identity. In addition, all samples tested negative for *mycoplasma* contamination (Supplementary Figure 1B).

### Validation of neural and mesenchymal lineage differentiation

**FIGURE 2:**
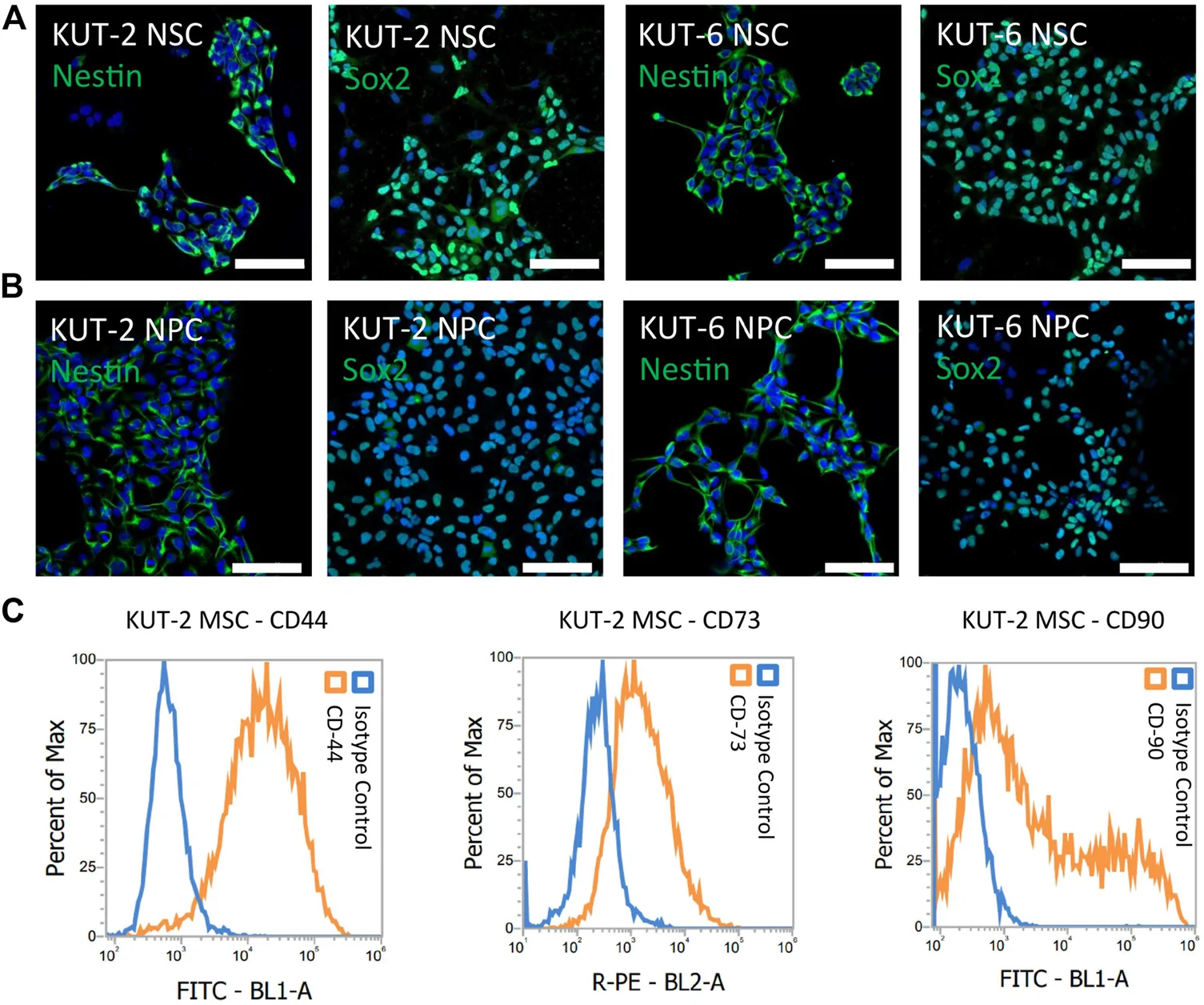
Characterization of NSCs, hNPCs, and MSCs. **(A)** Immunofluorescence staining of neuronal progenitor markers on NSCs; Nestin (green) and Sox2 (green), nuclei were counterstained with DAPI (blue). Scale bar size: 100 µM. **(B)** Immunofluorescence staining of hippocampal neuronal progenitors (hNPCs) for markers Nestin (green) and Sox2 (green), nuclei were counterstained with DAPI (blue). Scale bar size: 100 µM. **(C)** Validation of the expression of the MSC markers by CD44, CD73, and CD90 using flow cytometry in the KUT-2 line.

To evaluate lineage-specific differentiation potential, hiPSC lines were directed toward both neural and mesenchymal fates. Neural stem cells (NSCs) and hippocampal neural progenitor cells (hNPCs) were successfully generated from hiPSCs derived from both donors using two independent differentiation protocols (Figures 2A,B). Neural differentiation was confirmed by immunofluorescence staining for canonical neural progenitor markers. Cells derived from both hiPSC lines exhibited robust expression of Nestin and SOX2, indicating successful acquisition of neural progenitor identity (Figures 2A,B). The observed staining patterns and cellular morphology were consistent with neural stem/progenitor-like phenotypes, including elongated cellular structures and dense, progenitor-like populations. Both KUT-2 and KUT-6 lines demonstrated comparable marker expression profiles, suggesting reproducible and efficient neural induction across independent genetic backgrounds.

To validate the quality of the NSCs, we have further differentiated the resulting NSCs into neurons (Supplementary Figures 2A,C). We have confirmed that the neuronal cultures are functional, capable of both spontaneous electrical activity, which was validated by multielectrode array measurements (Supplementary Figures 2B,D,E,F), and ligand-induced responses, measured by calcium imaging (Supplementary Figures 2G,H). The resulting neurons are glutamatergic, therefore after the addition of 50 μM glutamate and in both cell lines we obtained a Ca-signal and most of the cells responded to the addition of glutamate.

These findings confirm that both hiPSC lines can generate functionally active neuronal populations, supporting their suitability for downstream applications involving neurobiological and pharmacological studies.

In parallel, mesenchymal differentiation capacity was assessed by directing hiPSCs toward an MSC-like lineage. Mesenchymal differentiation resulted in adherent, fibroblast-like cells displaying characteristic spindle-shaped morphology and plastic adherence, consistent with mesenchymal lineage commitment. Flow cytometric analysis demonstrated expression of canonical mesenchymal surface markers, including CD44 and CD90, with partial expression of CD73 (Figure 2C; Supplementary Figures 4A–C) and absence or low expression of hematopoietic and endothelial marker (CD45, CD34 and CD31) (Supplementary Figures 3A–C). Differentiation assays further supported mesenchymal identity: KUT-2-derived MSCs showed successful adipogenic and osteogenic differentiation (Supplementary Figures 3D–G). In contrast, KUT-6-derived cells showed limited differentiation capacity under the applied conditions, suggesting donor-dependent variability and incomplete mesenchymal maturation (Supplementary Figures 4D–G). Furthermore, they quickly entered a senescent state, so we could not perform cytotoxicity measurements on them. Together, these results establish a comparative platform for investigating lineage-dependent differences in cellular stress responses and drug sensitivity.

### Cytotoxicity patterns of KUT-2- and KUT-6-derived derivatives against DNA-damaging chemotherapeutics

**FIGURE 3:**
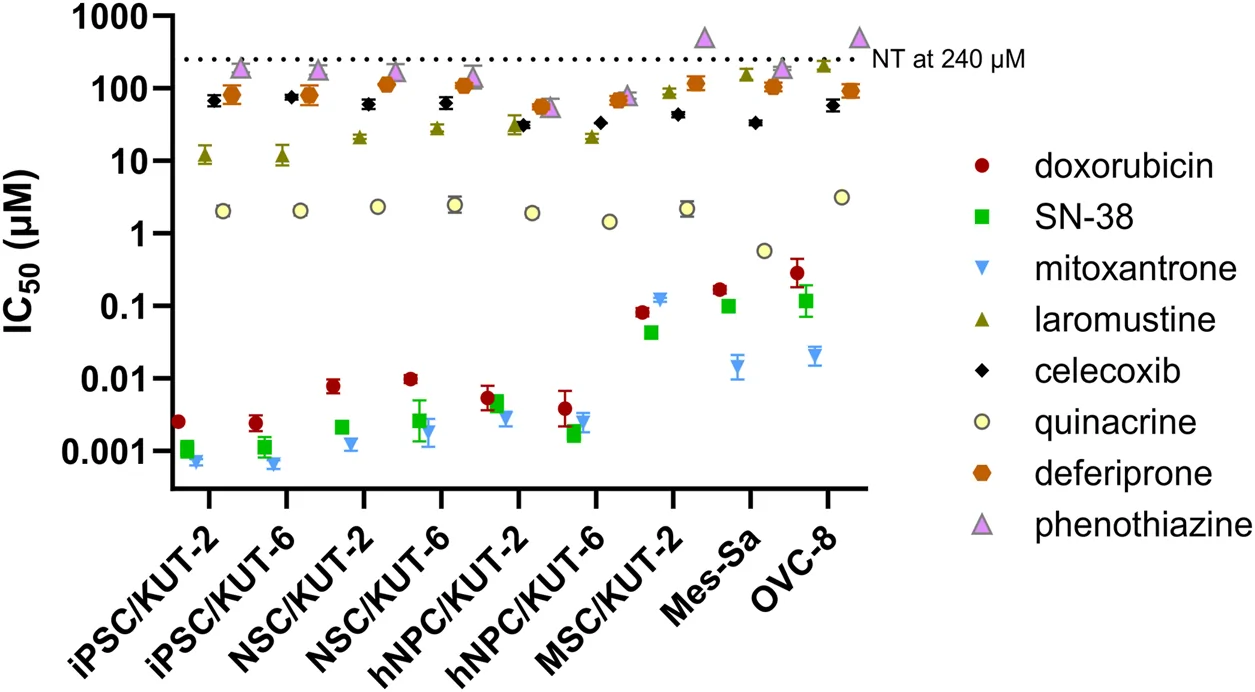
IC_50_ values with standard deviations of the anticancer agents and non-genotoxic drugs against the KUT-2 and KUT-6-derived cell lines, and against Mes-Sa and OVC-8 cancer lines. A dashed line at 240 µM indicates the highest tested concentrations, and compounds that did not reach the half-maximal inhibition against certain cell lines are presented above this line.

We evaluated the cytotoxicity effects of a panel of DNA-damaging anticancer agents across KUT-2 and KUT-6 hiPSCs as well as in their differentiated derivatives, including MSCs, NSCs, and hNPCs (Figure 3). Two-way ANOVA followed by Tukey’s *post hoc* test using the respective log IC_50_ values revealed that both KUT-2 and KUT-6 originated hiPSCs were the most sensitive, exhibiting the lowest IC_50_ values. hNPC and NSC derivatives displayed slightly lower sensitivity, with small but statistically detectable differences between them. MSC was consistently the most resistant derivative with a 1-2 orders of magnitude higher IC_50_ values compared to the other lines. These patterns were largely preserved across all tested compounds, suggesting that sensitivity is primarily dictated by cell type rather than compound-specific effects (Supplementary Table 3A). Differences in IC_50_ values between non-isogenic KUT-2- and KUT-6-derived cell types were minimal for the tested oncology drugs, further reinforcing that cell type is the main determinant of sensitivity among our cell lines.

When these four compounds were tested against the cancer cell lines OVC-8 and Mes-Sa, the IC_50_ values were similar to those measured in the MSC cell line: the MSC line was approximately only 2–3-fold more sensitive to doxorubicin, mitoxantrone, and laromustine, while it was several-fold more resistant to SN-38, even compared with the cancer cell lines. As a secondary observation, laromustine generally exhibited lower cytotoxicity against all cell lines, while doxorubicin, mitoxantrone and SN-38 were broadly comparable.

### Cytotoxicity patterns of KUT-2- and KUT-6-derived derivatives against non-genotoxic medicines

Cytotoxicity of four additional clinically used non-genotoxic drugs being repurposed for oncology applications in preclinical research (quinacrine, deferiprone, phenothiazine, and celecoxib), was also evaluated (Figure 3). The statistical analysis demonstrated that the primary determinant of drug sensitivity is the biological (differentiation) state of the cells, as most of the variation in IC_50_ values is explained by cell type rather than compound identity (Supplementary Table 3B). Post hoc comparisons confirmed this clear cell-type–dependent pattern. MSC, hiPSC, and NSC cells exhibited comparable IC_50_ values, with no significant differences among them, indicating similar levels of drug sensitivity within this group. In contrast, hNPC cells showed consistently and significantly lower IC_50_ values compared to all other cell types, reflecting a markedly higher sensitivity. This pattern was observed across both KUT-2 and KUT-6 origins, only minor numerical shifts were observed, most notably in hNPC cells (∼0.04 log units, that is roughly a 10% difference), which contributed slightly to overall variability but did not alter the general pattern.

When compared to the cancer cell lines Mes-Sa and OVC-8, the IC_50_ values were generally similar to those observed in MSC, hiPSC, and NSC populations, indicating comparable levels of drug sensitivity. Notably, OVC-8 cells exhibited the highest resistance, most prominently in response to phenothiazine, while responses to other compounds remained within the general range observed across cell types. Overall, these findings suggest that the cytotoxic effects of these non-genotoxic drugs are not cancer cell specific.

Compound identity had a secondary but detectable effect on cytotoxicity. Across all cell types, quinacrine exhibited the highest cytotoxicity, followed by celecoxib and deferiprone, while phenothiazine showed the lowest overall activity.

## Discussion

Several data-sets are available on the *in vitro* cytotoxicity of the active compounds examined in the present study; these reports have examined the effects of the substances using a wide range of cell lines, different methods, and exposure conditions, primarily in tumor cell lines (Supplementary Table 4). The advantage of the model system we have established is that it allows for the examination of different cell types with the same genetic background, thereby creating a common platform where the effects of individual substances can be determined relative to one another and which also provides the opportunity for combination of drug testing.

### Response to genotoxic stress: cell type is a more significant factor than biological sex or genetic background

Cellular drug sensitivity depends heavily on cell type and tissue of origin, driven by lineage-specific variations in metabolic state, cell cycle dynamics, DNA repair, and stress responses [58, 59]. Consequently, a single normal cell type cannot serve as a uniform control for lineage-specific cytotoxicity or predict clinical tissue toxicities, such as doxorubicin cardiotoxicity, cisplatin nephrotoxicity, or taxane neurotoxicity [60–62], whose underlying cellular mechanisms remain poorly defined.

To address this in a human-relevant NAM framework, we evaluated isogenic hiPSC, MSC, NSC, and hNPC lines from two donors (one female, one male) across compounds triggering diverse cytotoxic mechanisms. Distinct sensitivity patterns emerged across cell types, primarily driven by developmental state, proliferative activity, and lineage identity rather than donor sex, which yielded no pronounced differences. These findings align with prior work demonstrating differential toxicity across isogenic neural lineages [4]. Moreover, the demonstrated sensitivity of NPCs to cytotoxic agents could serve as a model of chemotherapy-related cognitive impairment („chemobrain”) mechanisms in future studies.

### DNA-damaging agents and iPSC hypersensitivity: a mechanism-specific pattern reflecting the genomic integrity strategy of pluripotent cells

One of the most consistent and mechanistically informative findings of the present study was the markedly elevated sensitivity of hiPSCs to DNA-damaging agents. Across all four compounds in this category—doxorubicin, mitoxantrone, SN-38, and laromustine—hiPSCs consistently showed the lowest IC_50_ values, while MSCs were the most resistant. Critically, this pattern was reproducible across both donor lines and was not compound-specific, but rather reflected a broader response to genotoxic stress.

This observation is consistent with a well-established body of literature demonstrating that human pluripotent stem cells (hPSCs) exhibit markedly higher sensitivity to DNA-damaging agents than their differentiated derivatives. Multiple studies have shown that hPSCs respond to genotoxic stress with rapid apoptosis, whereas differentiated progeny display greater resistance and preferentially activate cell cycle arrest and DNA repair pathways. This difference arises from the fundamentally distinct DNA damage response of pluripotent cells compared to differentiated cells.

Liu et al. demonstrated that human embryonic stem cells undergo significantly more rapid p53-dependent apoptosis following DNA damage than isogenic differentiated cells. This difference is not due to altered p53 function itself, but rather to the fact that pluripotent cells exist in a state of higher mitochondrial priming—meaning that even minor damage is sufficient to trigger cell death. The underlying basis is a shifted balance of pro- and anti-apoptotic proteins of the Bcl-2 family, which places these cells closer to the apoptotic threshold [63]. In a complementary study, Luo et al. showed that even at low doses of UV radiation, human pluripotent stem cells preferentially undergo apoptosis, while differentiated cells fail to mount a comparable apoptotic response, despite the pluripotent cells actually sustaining less UV-induced DNA damage than their differentiated counterparts [64]. Together, these findings indicate that the hypersensitivity of pluripotent cells to genotoxic stress is not simply a consequence of impaired DNA repair, but rather reflects a fundamentally different cellular decision-making strategy in response to genomic damage [65].

The differentiation-dependent loss of this hypersensitive apoptotic priming provides a mechanistic explanation for the gradient of drug sensitivity observed in our study across the hiPSC → NPC/MSC differentiation axis: as cells transition from an apoptosis-prone pluripotent phenotype toward a repair-tolerant differentiated state, a consistent biological gradient emerges that was well reflected in the IC_50_ patterns obtained with all four DNA-damaging agents. Moreover, this response was consistent across four distinct genotoxic classes—spanning topoisomerase I/II inhibitors, intercalators, and alkylating agents—these differences represent a fundamental, differentiation-dependent lowering of the apoptotic threshold rather than compound-specific toxicity.

### MSC resistance to cytotoxic agents: a consistent but mechanistically complex pattern

Among the cell types examined in the present study, MSCs derived from the KUT-2 donor line consistently displayed the highest resistance to cytotoxic compounds, with IC_50_ values one to two orders of magnitude higher than those observed in hiPSCs and NPC lineages. Notably, this resistance extended to comparisons with cancer cell lines: when the four DNA-damaging agents were tested against the OVC-8 and Mes-Sa tumor lines, the MSC line was only slightly more sensitive to doxorubicin, mitoxantrone, and laromustine, while it was even more resistant to SN-38. This pronounced tolerance aligns with previous studies showing naive MSCs frequently exhibit drug resistance comparable to or higher than fibroblasts [66, 67] and cancer cells [68]. Mechanistically, MSC resistance is non-universal and heavily context-dependent, governed by tissue source, compound mechanism of action [67, 68], and complex stress-response networks [69, 70]. For instance, Nicolay et al. demonstrated that bone marrow-derived MSCs resist cisplatin via high constitutive heat shock protein expression and suppressed p73/Bax/p21 pro-apoptotic signaling [66], yet remain uniquely vulnerable to bleomycin [67]. Taken together, our findings demonstrate that MSC resistance stems from overlapping survival strategies, including slow proliferation and attenuated apoptotic priming, and underscore that selecting an appropriate “normal cell” comparator is critical when evaluating therapeutic windows in drug discovery platforms.

### Lineage-specific vulnerability of neural progenitors

Our finding that hiPSC-derived neural progenitor cells (NPCs) exhibit greater sensitivity to non-genotoxic, metabolically active compounds than parental hiPSCs aligns with NPC dependency on lysosomal homeostasis and autophagic flux [71]. Compounds disrupting these pathways—such as quinacrine (lysosomal pH/autophagy inhibition) and deferiprone (iron homeostasis/mitochondrial stress)—uncovered a lineage-specific vulnerability that warrants further mechanistic study. Crucially, as NPCs represent a temporal window vulnerable to developmental and adult neurotoxicity [72], hippocampal NPCs (hNPCs) serve as sensitive models beyond conventional cytotoxicity endpoints. While CNS-penetrant compounds like phenothiazine directly exploit this vulnerability [73], our findings imply that non-permeable DNA-damaging agents could similarly trigger severe neural damage if the blood-brain barrier is compromised.

A conceptually similar protective role is fulfilled by the placenta during embryonic development. Like the BBB, the placental barrier limits fetal exposure to xenobiotics while maintaining nutrient and oxygen exchange [74]. However, placental transfer protection is incomplete, and numerous small molecules—particularly lipophilic, weakly basic, or transporter-favored compounds—can cross the placenta and reach the developing embryo [75]. This is particularly important because pluripotent cells of the early embryo are characterized by extremely high sensitivity to genotoxic stress, as demonstrated in our hiPSC experiments. Their rapid apoptotic response to DNA damage reflects the well-established “genomic guardian” strategy of pluripotent cells, in which preservation of genomic integrity is prioritized over survival. Consequently, hiPSCs provide a highly relevant *in vitro* model for embryotoxicity and teratogenicity assessment, particularly for compounds capable of crossing the placental barrier and interfering with early developmental processes.

The combination of hiPSC and hNPC models therefore provides complementary toxicological information across two critical developmental windows: early embryogenesis and early neurodevelopment. While hiPSCs serve as highly sensitive indicators of embryotoxic and genotoxic risk, hNPCs are particularly informative for detecting potential developmental neurotoxicity. This dual sensitivity strengthens the translational relevance of our model system and suggests that cytotoxicity testing based exclusively on rapidly proliferating tumor models may underestimate developmental and lineage-specific toxicity, whereas hiPSC-derived developmental models may provide a more physiologically relevant and predictive approach for human toxicity assessment.

### Limitations of the study

The core cytotoxic patterns observed — hiPSC hypersensitivity to DNA-damaging agents, hNPC sensitivity to metabolic stressors, and MSC resistance — were consistent across both donor lines, supporting the robustness of the main findings; nevertheless, replication in additional donor lines would be required to fully exclude donor-specific effects.

The experimental readout was based exclusively on cell viability and IC_50_ determination. While this approach allows for systematic quantitative comparison across cell types and compounds, it does not resolve the mechanistic fate of drug-exposed cells. Identical IC_50_ values may reflect fundamentally different cellular outcomes — including apoptosis, necrosis, quiescence, or senescence — which cannot be distinguished by viability assays alone. This is particularly relevant when interpreting the MSC data: as discussed above, the high IC_50_ values in MSCs may reflect a shift toward drug-induced quiescence rather than true resistance in the conventional pharmacological sense [69]. Complementary mechanistic endpoints — including caspase activation, cell cycle analysis, senescence markers, ROS quantification, and lysosomal or mitochondrial function assays — would be needed to fully characterize the cell fate consequences of drug exposure in each lineage.

The cell type panel was intentionally restricted to hiPSCs, MSCs, and NPC lineages, representing two of three embryonic germ layers. The inclusion of a third germ layer derivative — most relevantly hiPSC-derived cardiomyocytes — would have further extended the comparative framework. At the same time, cardiotoxicity assessment in hiPSC-derived cardiomyocytes is a well-established and extensively studied area [76, 77], that certain compound-specific toxicities — in particular, the cardiotoxic potential of doxorubicin — remain unaddressed in the present dataset.

We still suggest that the presented screening methods are superior in several aspects as compared to studies performed in fibroblast-cancer cell duets. This is especially true regarding the less differentiated, stem cell-like cells, potentially present in inhomogeneous tissue samples with greatly different drug sensitivities. *In vivo* animal studies could reinforce our conclusions, when undifferentiated, stem cell like cells can be separately examined.

Taken together, while these limitations should be considered in interpreting the findings, the main cytotoxic patterns identified in this study — genotoxic hypersensitivity in hiPSCs, metabolic vulnerability in hippocampal NPCs, and broad cytotoxic resistance in MSCs — were internally consistent, reproducible across both donor lines, and mechanistically coherent with the existing literature. These features support the biological relevance of the model and the robustness of the principal conclusions.

## Data Availability

The raw data supporting the conclusions of this article will be made available by the authors, without undue reservation.

## Glossary

3R: replace, reduce, and refine animal models
ADR: Adverse drug reactions
AFP: α-fetoprotein
Akt: Protein kinase B
ATCC: American Type Culture Collection
BBB: Blood-brain-barrier
BSA: Body surface area
CNS: Central nervous system
EB: Embryoid body
EMA: European Medicines Agency
ERK1/2: extracellular signal-regulated kinase
FDA: Food and Drug Administration
hiPSC: Human induced pluripotent stem cell
hNPC: Hippocampal neural progenitor cell
hPSC: Human pluripotent stem cell
IMRWG3R: International Medicines Regulators’ Working Group on 3Rs
MAPK: Mitogen-activated protein kinase
MSC: Mesenchymal stem cell-like cell
mTOR: mechanistic Target of Rapamycin
MTT: 3-(4,5-dimethylthiazol-2-yl)-2,5-diphenyl tetrazolium bromide
NAM: New approach methodologies
NF-κB: Nuclear factor-κB
NIH NCI DTP: National Cancer Institute Developmental Therapeutics Program of the National Institute of Health
NSC: Neural stem cell
Pax6: paired box 6
PDK1: phosphoinositide-dependent kinase 1
PGE2: Prostaglandin E2
SI: Selectivity index
SMA: α-smooth muscle actin
Sox1: Sry-box transcription factor 1
Sox2: Sry-box transcription factor 2
SSEA4: Stage-specific embryonic antigen-4
STR: Short tandem repeat
TUB: β-III tubulin
