# Supplementary material for: Beyond animal models: using human pluripotent stem cell-derived stem and progenitor cells to predict cytotoxicity in normal tissues
Source: Pathol Oncol Res. 2026 Aug 20;32:1612490. doi: 10.3389/pore.2026.1612490 (PMC13538076; doi:10.3389/pore.2026.1612490)
Supplement: Supplementary file 1 [file DataSheet1.pdf]

Supplementary materials

Mycoplasma test

Mycoplasma detection was performed with MyxoAlertTM Mycoplasma Detection Kit according to the manufacturer's instructions. To detect the bioluminescence PerkinElmer (Shelton, CT, USA) VICTOR X3 Multilabel Reader was used.

Neuronal differentiation

The NSCs were seeded on eight-well chambers previously coated with poly-ornithine/laminin. On the day after seeding, the Neuronal Expansion Media was switched to a Neuronal Differentiation media containing Neurobasal Plus, supplemented with 2% B-27 plus, 1% Glutamax; 0,5% CultureOne Supplement, 0.1% ascorbic acid and 1% Antibiotic-Antimycotic.

Ca-imaging

The hNPCs or NSCs were differentiated into what for 4 weeks. Before measurement, the cells were treated with 1.0 µM Fluo-4 AM in a serum-free culture medium for 30 min at 37 °C. The medium was removed, and the measurements were taken in Hank's balanced salt solution (HBSS) supplemented with 20 mM Hepes (pH = 7.4) and 0.9 mM $MgCl_2$. The ligand, glutamate, concentration was 50 µM. All experiments were performed at 37 °C using an Ibidi Heating System. Calcium signal measurements were carried out by acquisition of time-lapse images on a Zeiss LSM 900. Fluorescence images were acquired between 505 and 525 nm at 488 nm excitation. Image analysis was carried out with ImageJ software. The fluorescence data were normalized to the baseline (F0) fluorescence levels before glutamate admission using the F/F0 formula, where F is the Ca^{2+} signal intensity at a given time point and F0 is the average of the 50 time points before glutamate admission.

MEA measurements

The NSCs were differentiated into neurons for 5 weeks on 6-well MEA200/30iRTi (Multi Channel Systems, Reutlingen, Germany) poly-ornithine-coated chips, as described above. Extracellular electrophysiological recordings were collected using the MEA2100-System (Multi Channel Systems, Reutlingen, Germany). Recordings were carried out at 37◦ at a sampling rate of 20 kHz. The raw recordings were filtered and visualized using MEA-NAP pipeline (version: 1.11.0) (1).

(A)

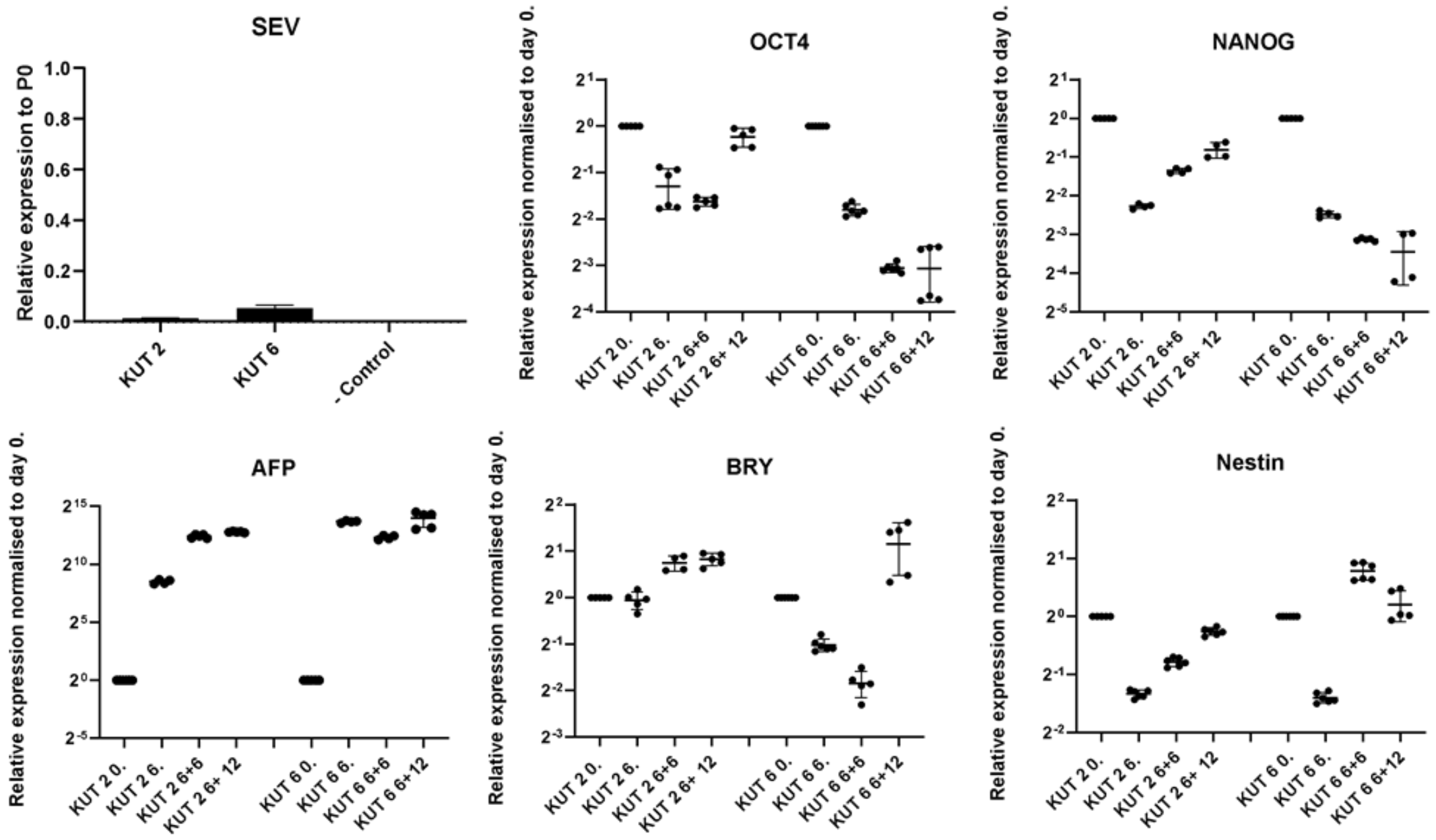

(B)

	1st read (CPS)	2nd read (CPS)	2nd read / 1st read
iPSC KUT2-D6	303	183	0.604
iPSC KUT6-C6	399	229	0.574
Positive control	290	457	1.576

Supplement Figure 1. (A) Sendai virus clearance by RT-PCR; Expression of pluripotency (Oct4 and Nanog) and differentiation markers (endoderm-AFP, mesoderm- Brachyury and ectoderm-Nestin) during spontaneous differentiation at EB day 0, day 6, day 6+6, and 6+12 by RT-PCR. **(B)** Mycoplasma Detection: The values are expressed in Counts Per Second (CPS). The ratio of the two reads (2nd read / 1st read) determines whether the sample is contaminated (>1.2) or negative for mycoplasma (<0.9).

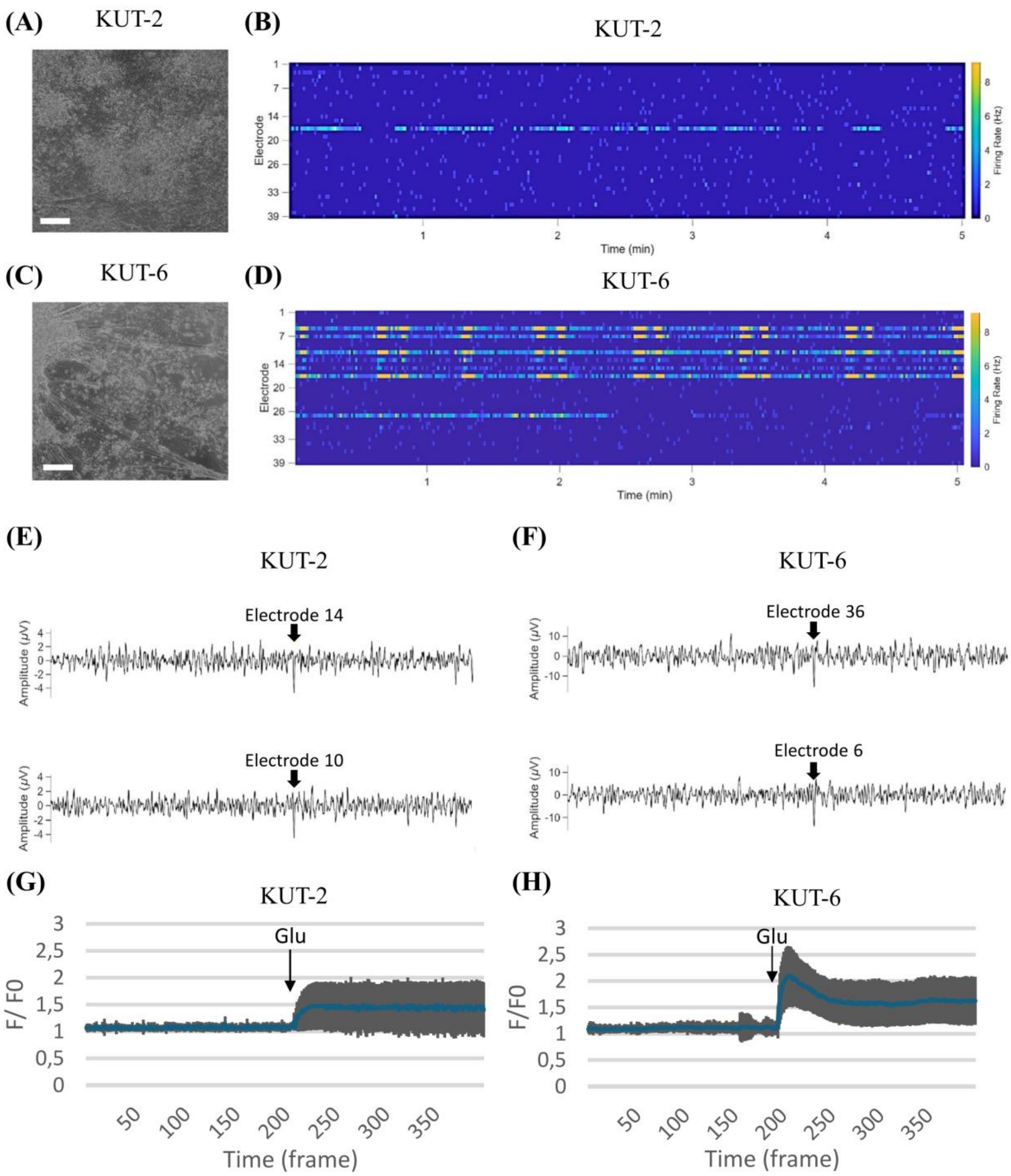

Supplementary Figure 2. Neuronal morphology and functional validation. (**A**) Representative picture of **KUT-2** neuronal morphology at 2 weeks of differentiation of NSCs. (**B**) Representative raster plot on neuronal activity of **KUT-2** after 27 days of differentiation. Each row represents an electrode, and each vertical line represents a spike; the measurement time is five minutes. (**C**) Representative picture **KUT-6** neuronal morphology at 2 weeks of differentiation of NSCs. (**D**) Representative raster plot on neuronal activity of **KUT-6** after 33 days of differentiation. Each row represents an electrode, and each vertical line represents a spike; the measurement time is five minutes. **(E)** Sample voltage traces from single electrodes after 27 days of differentiation of the **KUT-2** cell line. The arrow indicates a spike detected by both thr5 and bior1.5 methods **(F)** Sample voltage traces from single electrodes after 33 days of differentiation of the **KUT-6** cell line. The arrow indicates a spike detected by both thr5 and bior1.5 methods **(G)** Ligand-induced response to glutamate (50 µM) after 4 weeks of differentiation on **KUT-2** cultures. **(H)** Ligand-induced response to glutamate (50 µM) after 4 weeks of differentiation, on **KUT-6** cultures. Changes in fluorescence were described by

F/F0, where F is the Ca^{2+} signal intensity at a given time point, and F0 is the average of the 50 time points before glutamate admission.

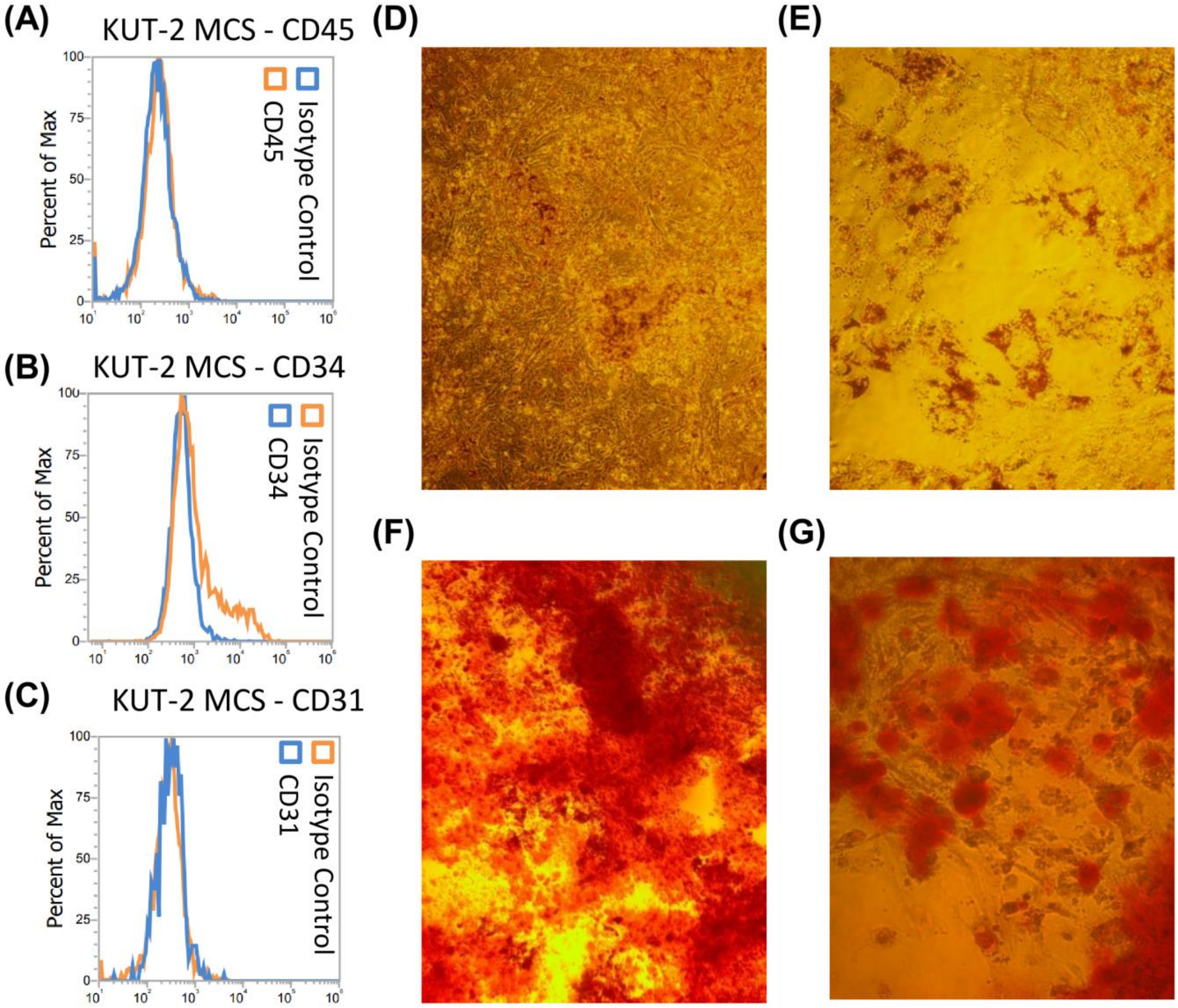

Supplementary Figure 3. Validation of the MSC-like cells differentiated from KUT-2 hiPSC line – **(A-C)** The established line is negative for the hematopoietic markers CD45 **(A)**, CD34 **(B)** and endothelial marker CD31**(C)**. (**D-E**) Confirmation of adipogenesis by Oil Red O staining on day 22 of differentiation; **(D)** 10x magnification; **(E)** 40x magnification. (**F-G)** Confirmation of osteogenesis on day 22 of differentiation by alizarin red staining. **(F)** 4x **(G)** 20x. magnification.

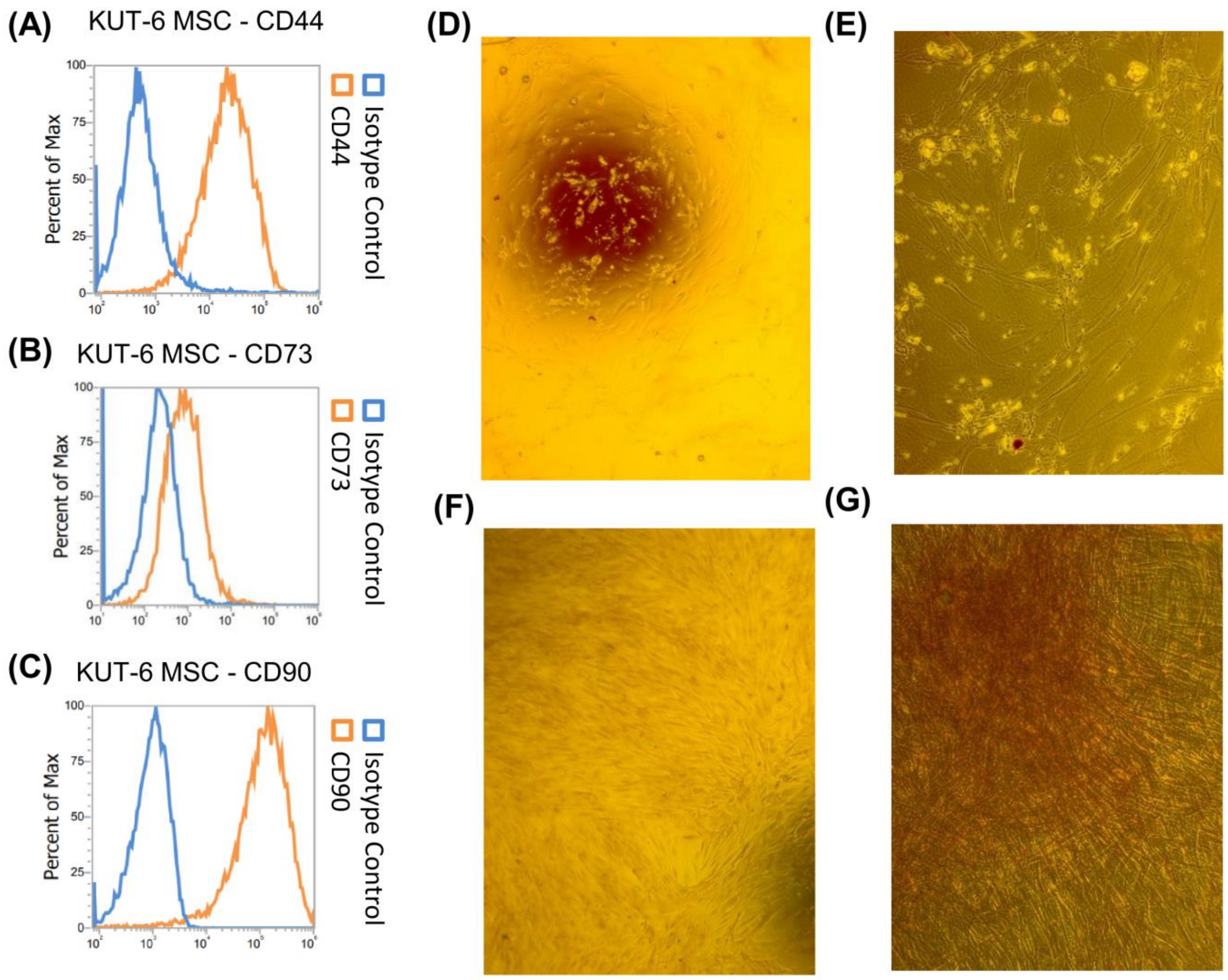

Supplementary Figure 4: Validation of MSC differentiation and markers on KUT-6-derived MSC-like cells: Expression of MSC markers **(A)** CD44, **(B)** CD73 and **(C)** CD90. **(D-E)** Oil Red O staining on KUT-6 after 22 days of adipogenic differentiation; **D)** 4x magnification; **C)** 10x magnification. (**F-G)** Alizarin red staining on KUT-6 after 22 days of osteogenic differentiation **D)** 4x and **E)** 10x magnification.

Supplementary Table 1. Summary table of substances used, their known effects, and available data on their passage through the placenta and the blood-brain barrier. Compounds marked with “#” are anticancer drugs, while those marked with “*” are currently being tested for use in oncology. Abbreviation: BSA: Body surface area. BBB: blood-brain-barrier, FDA: Food and Drug Administration.

**Compound**	**Application**	**Mechanism of action**	**Ability to cross BBB**	**Ability to cross the placenta**	**Therapeutic dose (mg/BSA/Cmax) /**
Doxorubicin #	Chemotherapy drug, non-cancer specific	Intercalation of DNA, inhibition of topoisomerase II (2)	Limited due to active efflux (3) substrate of ABCG2 (4)	Accumulate in the placenta, limited data available (5)	Administration 12-90 mg/m^2, Plasma level around 1-20 μM (6);(7)
Mitoxantrone #	Chemotherapy drug	Topoisomerase II inhibition, disturbs DNA synthesis and repair (8)	Limited due to active efflux (9, 10)	Limited due to active efflux (11)	Administration 2-14 mg/m^2, depends on the cancer type and the patient status (12)
SN-38 #	Chemotherapy drug	Topoisomerase I inhibition (13)	Limited due to active efflux substrate of ABCG2 (14) and ABCB1 (15)	Limited due to active efflux (14)	Administration: 20 - 29 mg/m^2 CPT-11 Peak plasma concentration: 1.2 μM. Administration of irinotecan 100 to 350 mg/m^2, maximum plasma concentration 1-10 mg/L, while SN-38 100 fold lower (16)
Laromustine # (101M)	Chemotherapy (pro)drug, myeloid leukemia (17)	DNA Alkylation (18)	Confirmed in rat model (19).Tested in Phase I for pediatric brain tumor (20)and Phase II for glioblastoma multiforme	No evidence	Administration: 220-600 mg/m^2 for Refractory Leukemia Peak plasma concentration: 4.8+-2.6- 11.4+-3.9 μM (21)
Celecoxib *	Cox2 inhibitor NSAID	Cox2 inhibitor (22)	Yes, validated in human (23)	Yes, based on structure, no direct information about transplacental transfer (24)	100-400 mg /day (25)

Quinacrine *	Antimalarial drug, also used in lupus erythematosus treatment	DNA intercalation, lysosomotropic (26, 27)	Limited due to active efflux (28)	No data available (FDA)	250–500 mg daily, maximum 4 mg/kg lean bodyweight (29).
Deferiprone *	Used to treat chronic iron overload caused by frequent blood transfusions (for example thalassemia major)	Iron chelator (30)	Yes, validated in rat (31)	No direct evidence, but based on animal models embryotoxic (32)	Recommended dose is 50-75 mg / kg / day (max 100 mg) (32); peak plasma concentration is 100 µmol/L (33).
Phenothiazine *	Neurolepticum, chemotherapy drug (34)	Blockage of dopamine D1 and D2, muscarinic, histamine H1 and serotonergic 5-HT2; cell membrane disruption (35)	Able to cross the blood brain barrier (36)	Yes, based on structure, confirmed for derivatives. (37)	Information only available for derivatives

Supplementary table 2. List of reagents including supplier and catalogue number.

Reagent	Cat. number	Supplier
**Media and supplements**		
StemPro-34 medium	10639011	Thermo Fisher Scientific
GlutaMax	35050-061	
β-mercaptoethanol	31350-010	
Antibiotic-Antimycotic	15240062	
IL-3	200-06-20UG	
IL-6	200-03-10UG	
FLT-3	300-19-10UG	
SFC	300-07-10UG	
KO-DMEM	10829-018	
KO-Serum Replacement	10828028	
NEAA	11140-035	
bFGF	13256029	
DMEM medium	41965039	
ES tested FBS	16141079	
DMEM/F-12, 2 mM GlutaMAX™	35050-061	
N2	12587010	
B27	17502048	
laminin	23017015	
Neural Induction Medium	A1647801	
AdvancedTM DMEM⁄F-12	12634	
Neurobasal® Medium	A1647801	
Neuronal Induction Supplement	A1647801	
StemPro Accutase	A1110501	
Matrigel	354277	Corning
mTeSR1	85850	STEMCELL Technologies
ReLeSR	5872	
bovine serum albumin (BSA)	A9418-50G	Sigma-Aldrich
**Assay reagents**		
DAPI	D1306	Thermo Fisher Scientific
Propidium-Iodide	P1304MP	
CytoTune-iPS 2.0 Sendai Reprogramming Kit	A16517	
TaqMan® gene expression assays	4331182	

SEV (AB_2760502)	A13640	
BRY (Hs00610080_m1)		
OCT4 (Hs00999632_g1)		
GATA4 (Hs00171403_m1)		
AFP (Hs00173490_m1)		
NANOG (Hs02387400_g1)		
Nestin (Hs04187831_g1)		
RPLP0 (Hs9999902_m1)		
PrestoBlue	A13262	
Human Mesenchymal Stem Cell Functional Identification Kit	SC006	Biotechne
MyxoAlertTM Mycoplasma Detection Kit	LT07-318	Lonza, Basel, Switzerland
**Chemical compounds**		
Deferiprone	379409	Merck
celecoxib	SML3031	
phenothiazine	P14831	
quinacrine	551850	
SN-38	H0165	
doxorubicin	D2975000	
Mitoxantrone	24343907	Molekula Ltd.
Laromustine*		Servier Research Institute of Medicinal Chemistry
**antibodies**		
PE anti-human SSEA-4	RD-FAB1435A-100	R&D Systems
Isotype control for SSEA4	1C007A	R&D Systems
PE anti-human CD73	550257	Becton Dickinson
PE anti-human CD31	555476	
FITC anti-human CD34	345801	
FITC anti-human CD44	555478	
PE anti-human CD45	555483	
anti-human CD90	550402	
PE Mouse IgG1	345816	
APC Mouse IgG2b	555745	
FITC Rat IgG2b	sc-2835	Santa Cruz
secondary goat-anti-mouse-IgG2b Alexa-488	A21141	Invitrogen
Alexa Fluor 647 goat anti-mouse IgG	A11029	Thermo Fisher Scientific

Alexa Fluor 594 donkey anti-goat IgG	A11012	Thermo Fisher Scientific
OCT3/4	SC-5279	Santa Cruz
NANOG	AF1997	R&D Systems
AFP	A8452	Sigma-Aldrich
α-SMA	ab7817	Abcam
βIII Tubulin	RD-MAB1195	R&D Systems
SOX1	AF3369	Bio-Techne
SOX2	MAB2018	R&D Systems
PAX6	PRB-278P-100	BioLegend
Nestin	ab92391	Abcam

<u>Suppliers headquarters:</u>

Thermo Fisher Scientific (Waltham, MA, USA)
Corning, New York, NY, USA
STEMCELL Technologies, Vancouver, BC, Canada
Sigma-Aldrich, St. Louis, MO, USA
Biotechne, Minneapolis, MN, USA
Merck, Sigma-Aldrich, Darmstadt, Germany
Molekula Ltd., Newton Aycliffe, UK
* kindly provided by Servier Research Institute of Medicinal Chemistry (SRIMC), Budapest, Hungary
R&D Systems, Minneapolis, United States
Becton Dickinson, New Jersey, United States
Santa Cruz Santa Cruz, USA
Invitrogen, Carlsbad, California, USA
Abcam, Cambridge, UK
BioLegend, San Diego, CA, USA

Supplementary table 3. ANOVA summary statistics for (A) chemotherapeutic (B) non-chemotherapeutic drug response across cell types. SS, sum of squares; DF, degrees of freedom; MS, mean square; F, F statistic; DFn, numerator degrees of freedom; DFd, denominator degrees of freedom. "% of total variation" indicates the proportion of overall variance explained by each factor in the two-way ANOVA model. Cell identity was used as the row factor, while compound identity was used as the column factor. Cell identity corresponds to the analyzed cell lines, while compound identity represents the tested genotoxic or non-genotoxic agents. Interaction denotes the combined effect of cell identity and compound identity. Residual represents unexplained variance within the model. Statistical significance was determined using ordinary two-way ANOVA with $\alpha = 0.05$.

(A)

Source of variation	SS (Type III)	DF	MS	F (DFn, DFd)	% of total variation	P value
Cell identity (row factor)	296.4	3	98.79	F(3,81) = 5029	90.0	<0.0001
Compound identity (column factor)	26.4	6	4.40	F(6,81) = 223.8	8.0	<0.0001
Interaction	3.6	18	0.20	F(18,81) = 10.32	1.1	<0.0001
Residual	1.6	81	0.02	—	—	—

(B)

Source of variation	SS (Type III)	DF	MS	F (DFn, DFd)	% of total variation	P value
Cell identity (row factor)	55.6	3	18.54	F(3,80) = 2259	95.8	<0.0001
Compound identity (column factor)	1.5	6	0.26	F(6,80) = 31.43	2.7	<0.0001
Interaction	0.7	18	0.04	F(18,80) = 4.598	1.2	<0.0001
Residual	0.7	80	0.01	—	—	—

Supplementary Table 4: Comparison of the known cytotoxic effects of the tested substances in *in vitro* systems and the average IC_{50} values measured on KUT-2; KUT-6 respectively, in this article.

Compound	Average IC_{50} [μM]	Effective dose
Doxorubicin	iPSC: 0.0025; 0.0024<br>NSC: 0.0078; 0.0098<br>hNPC: 0.0054; 0.0038<br>MSC: 0.082<br>Mes-Sa / OVC-8:<br>0.17; 0.28	On cancer cell lines the effective IC50 depends on the formulation and cell line: IC_{50} around 11 μM-218 μM (38). On mural neuronal cells decreased cell viability after 24 hours even at 0.1-0.5 μM (3).
Mitoxantrone	iPSC: 0.0011; 0.0011<br>NSC: 0.0021; 0.0026<br>hNPC: 0.0045; 0.0017<br>MSC: 0.043<br>Mes-Sa / OVC-8:<br>0.10; 0.12	Significantly decrease PDAC tumor cells viability in spheroid assay from 5 μg /ml after 4 days (39). Less than 100 ng can decrease P388/S leukemia cell line viability (40).
SN-38	iPSC: 0.0007; 0.0006<br>NSC: 0.0012; 0.0018<br>hNPC: 0.0028; 0.0025<br>MSC: 0.121<br>Mes-Sa /OVC-8:<br>0.014; 0.020	1 μmol/L decreased the cells viability significantly after 48 hours in colorectal cancer cells (41). Breast cancer cells MCF-7, fibrosarcoma cells HT1080 and hepatoma cells HepG2 cells 0.1-0.5 μg / mL decreased the cell's viability by 50% after 48 hours (42).
Laromustine (101M)	iPSC: 12,2; 12,0<br>NSC: 21.4; 28.3<br>hNPC: 31.4, 21.6<br>MSC: 90.2<br>Mes-Sa /OVC-8:<br>155.8; 208.9	LC50: 5.74 μM of the active form 90CE on HL-60 cells after 3 days of treatment (43).
Celecoxib	iPSC: 67.2; 75.4<br>NSC: 60.3; 62.4<br>hNPC: 31.1; 33.3<br>MSC: 43.4<br>Mes-Sa /OVC-8:<br>33.3; 58.2	On immortalized cell lines (LLC. HCA-7 and HCT-15) 20 μM for 12 hours reduced the viability (44). On human lung cancer lines, after 72 hours A549. GLC82 and SW1573 IC_{50}-s were 14.7. 10.6. and 18.6 μmol/L (45).

Quinacrine	iPSC: 2.03; 2.04<br>NSC: 2.33; 2.49<br>hNPC: 1.91; 1.44<br>MSC: 2.17<br>Mes-Sa / OVC-8: 0.57; 3.15	Tested in combination on HL-60 and MV4-11 cell lines. Combination with cytarabine is more effective than alone both *in vitro* and *in vivo* (46). Induces K562 cell apoptosis (47). High toxicity above 5 μM in ScN2a mouse cell line (48). In combination with temozolomide decrease the cells viability in glioblastoma stem cells (49).
Deferiprone	iPSC: 81.8; 80.4;<br>NSC: 112.8; 109.0;<br>hNPC: 55.6; 68.4<br>MSC: 117.5<br>Mes-Sa / OVC-8: 104.1; 91.7	IC_{50} in liquid myeloid culture system 150-172 μM (50). On breast cancer lines MCF7 and T47D after 5 days IC_{50} was between 75 and 100 μM. while the tested fibroblasts and epithelial cells were more resistant (51). The IC_{50} on Mes-Sa and Mes-Sa/B1 cells was 121 μM and 100 μM respectively (52).
Phenothiazine	iPSC: 190.5; 179.9<br>NSC: 170.8; 142.9<br>hNPC: 54.3; 79.4<br>MSC: >240<br>Mes-Sa / OVC-8: 188.4; >240	Phenothiazine was not toxic up to 125 μM in a 24h citotoxicity assay against hepatoma tissue culture (HCT) cells (53).<br>Information about derivatives: Thioridazine 15 μM for 24 h on K562 cells (54).

REFERENCES

1. Sit TPH, Feord RC, Dunn AWE, Chabros J, Oluigbo D, Smith HH, Burn L, Chang E, Boschi A, Yuan Y, Gibbons GM, Khayat-Khoei M, De Angelis F, Hemberg E, Hemberg M, Lancaster MA, Lakatos A, Eglen SJ, Paulsen O, Mierau SB. MEA-NAP: A flexible network analysis pipeline for neuronal 2D and 3D organoid multielectrode recordings. Cell reports methods. 2024;4(11):100901.
2. Kik K, Studzian K, Wasowska-Lukawska M, Oszczapowicz I, Szmigiero L. Cytotoxicity and inhibitory properties against topoisomerase II of doxorubicin and its formamidine derivatives. Acta biochimica Polonica. 2009;56(1):135-42.
3. Kaminska K, Cudnoch-Jedrzejewska A. A Review on the Neurotoxic Effects of Doxorubicin. Neurotoxicity research. 2023;41(5):383-97.
4. Stacy AE, Jansson PJ, Richardson DR. Molecular pharmacology of ABCG2 and its role in chemoresistance. Molecular pharmacology. 2013;84(5):655-69.
5. Ryu RJ, Eyal S, Kaplan HG, Akbarzadeh A, Hays K, Puhl K, Easterling TR, Berg SL, Scorsone KA, Feldman EM, Umans JG, Miodovnik M, Hebert MF. Pharmacokinetics of doxorubicin in pregnant women. Cancer chemotherapy and pharmacology. 2014;73(4):789-97.
6. Gewirtz DA. A critical evaluation of the mechanisms of action proposed for the antitumor effects of the anthracycline antibiotics adriamycin and daunorubicin. Biochemical pharmacology. 1999;57(7):727-41.
7. Frenay M, Milano G, Renee N, Pons D, Khater R, Francois E, Thyss A, Namer M. Pharmacokinetics of weekly low dose doxorubicin. European journal of cancer & clinical oncology. 1989;25(2):191-5.

8. Evison BJ, Sleebs BE, Watson KG, Phillips DR, Cutts SM. Mitoxantrone, More than Just Another Topoisomerase II Poison. Medicinal research reviews. 2016;36(2):248-99.
9. Agarwal S, Hartz AM, Elmquist WF, Bauer B. Breast cancer resistance protein and P-glycoprotein in brain cancer: two gatekeepers team up. Current pharmaceutical design. 2011;17(26):2793-802.
10. Lee YJ, Kusuhara H, Jonker JW, Schinkel AH, Sugiyama Y. Investigation of efflux transport of dehydroepiandrosterone sulfate and mitoxantrone at the mouse blood-brain barrier: a minor role of breast cancer resistance protein. The Journal of pharmacology and experimental therapeutics. 2005;312(1):44-52.
11. Kolwankar D, Glover DD, Ware JA, Tracy TS. Expression and function of ABCB1 and ABCG2 in human placental tissue. Drug metabolism and disposition: the biological fate of chemicals. 2005;33(4):524-9.
12. Faulds D, Balfour JA, Chrisp P, Langtry HD. Mitoxantrone. A review of its pharmacodynamic and pharmacokinetic properties, and therapeutic potential in the chemotherapy of cancer. Drugs. 1991;41(3):400-49.
13. Kumar S, Sherman MY. Resistance to TOP-1 Inhibitors: Good Old Drugs Still Can Surprise Us. International journal of molecular sciences. 2023;24(8).
14. Bates SE, Medina-Perez WY, Kohlhagen G, Antony S, Nadjem T, Robey RW, Pommier Y. ABCG2 mediates differential resistance to SN-38 (7-ethyl-10-hydroxycamptothecin) and homocamptothecins. The Journal of pharmacology and experimental therapeutics. 2004;310(2):836-42.
15. Tagen M, Zhuang Y, Zhang F, Harstead KE, Shen J, Schaiquevich P, Fraga CH, Panetta JC, Waters CM, Stewart CF. P-glycoprotein, but not multidrug resistance protein 4, plays a role in the systemic clearance of irinotecan and SN-38 in mice. Drug metabolism letters. 2010;4(4):195-201.
16. Chabot GG. Clinical pharmacokinetics of irinotecan. Clinical pharmacokinetics. 1997;33(4):245-59.
17. Giles F, Vey N, DeAngelo D, Seiter K, Stock W, Stuart R, Boskovic D, Pigneux A, Tallman M, Brandwein J, Kell J, Robak T, Staib P, Thomas X, Cahill A, Albitar M, O'Brien S. Phase 3 randomized, placebo-controlled, double-blind study of high-dose continuous infusion cytarabine alone or with laromustine (VNP40101M) in patients with acute myeloid leukemia in first relapse. Blood. 2009;114(19):4027-33.
18. Pigneux A. Laromustine, a sulfonyl hydrolyzing alkylating prodrug for cancer therapy. IDrugs : the investigational drugs journal. 2009;12(1):39-53.
19. Mao J, Xu Y, Wu D, Almassain B. Pharmacokinetics, mass balance, and tissue distribution of a novel DNA alkylating agent, VNP40101M, in rat. AAPS pharmSci. 2002;4(4):E24.
20. Gururangan S, Turner CD, Stewart CF, O'Shaughnessy M, Kocak M, Poussaint TY, Phillips PC, Goldman S, Packer R, Pollack IF, Blaney SM, Karsten V, Gerson SL, Boyett JM, Friedman HS, Kun LE. Phase I trial of VNP40101M (Cloretazine) in children with recurrent brain tumors: a pediatric brain tumor consortium study. Clinical cancer research : an official journal of the American Association for Cancer Research. 2008;14(4):1124-30.
21. Giles F, Thomas D, Garcia-Manero G, Faderl S, Cortes J, Verstovsek S, Ferrajoli A, Jeha S, Beran M, Koller C, Andreeff M, Cahill A, Clairmont C, Sznol M, Kantarjian H. A Phase I and pharmacokinetic study of VNP40101M, a novel sulfonylhydrazine alkylating agent, in patients with refractory leukemia. Clinical cancer research : an official journal of the American Association for Cancer Research. 2004;10(9):2908-17.
22. Wen B, Wei YT, Mu LL, Wen GR, Zhao K. The molecular mechanisms of celecoxib in tumor development. Medicine. 2020;99(40):e22544.

23. Dembo G, Park SB, Kharasch ED. Central nervous system concentrations of cyclooxygenase-2 inhibitors in humans. Anesthesiology. 2005;102(2):409-15.
24. Boubred F, Vendemmia M, Garcia-Meric P, Buffat C, Millet V, Simeoni U. Effects of maternally administered drugs on the fetal and neonatal kidney. Drug safety. 2006;29(5):397-419.
25. Fleischmann R, Tannenbaum H, Patel NP, Notter M, Sallstig P, Reginster JY. Long-term retention on treatment with lumiracoxib 100 mg once or twice daily compared with celecoxib 200 mg once daily: a randomised controlled trial in patients with osteoarthritis. BMC musculoskeletal disorders. 2008;9:32.
26. Eriksson A, Chantzi E, Fryknas M, Gullbo J, Nygren P, Gustafsson M, Hoglund M, Larsson R. Towards repositioning of quinacrine for treatment of acute myeloid leukemia - Promising synergies and in vivo effects. Leukemia research. 2017;63:41-6.
27. Eriksson A, Osterroos A, Hassan S, Gullbo J, Rickardson L, Jarvius M, Nygren P, Fryknas M, Hoglund M, Larsson R. Drug screen in patient cells suggests quinacrine to be repositioned for treatment of acute myeloid leukemia. Blood cancer journal. 2015;5(4):e307.
28. Ghaemmaghami S, Ahn M, Lessard P, Giles K, Legname G, DeArmond SJ, Prusiner SB. Continuous quinacrine treatment results in the formation of drug-resistant prions. PLoS pathogens. 2009;5(11):e1000673.
29. Wozniacka A, McCauliffe DP. Optimal use of antimalarials in treating cutaneous lupus erythematosus. American journal of clinical dermatology. 2005;6(1):1-11.
30. Dexter DT, Statton SA, Whitmore C, Freinbichler W, Weinberger P, Tipton KF, Della Corte L, Ward RJ, Crichton RR. Clinically available iron chelators induce neuroprotection in the 6-OHDA model of Parkinson's disease after peripheral administration. Journal of neural transmission. 2011;118(2):223-31.
31. Ward RJ, Dexter D, Florence A, Aouad F, Hider R, Jenner P, Crichton RR. Brain iron in the ferrocene-loaded rat: its chelation and influence on dopamine metabolism. Biochemical pharmacology. 1995;49(12):1821-6.
32. Jamuar SS, Lai AH. Safety and efficacy of iron chelation therapy with deferiprone in patients with transfusion-dependent thalassemia. Therapeutic advances in hematology. 2012;3(5):299-307.
33. Galanello R. Deferiprone in the treatment of transfusion-dependent thalassemia: a review and perspective. Therapeutics and clinical risk management. 2007;3(5):795-805.
34. Mehrabi SF, Elmi S, Nylandsted J. Repurposing phenothiazines for cancer therapy: compromising membrane integrity in cancer cells. Frontiers in oncology. 2023;13:1320621.
35. Zou X, Xie B. Therapeutic Mechanisms of Phenothiazine Drugs: A Mini-Review of Advances in Cancer Treatment and Antibiotic Resistance. Iranian journal of pharmaceutical research : IJPR. 2025;24(1):e157923.
36. Sudeshna G, Parimal K. Multiple non-psychiatric effects of phenothiazines: a review. European journal of pharmacology. 2010;648(1-3):6-14.
37. Hammond JE, Toseland PA. Placental transfer of chlorpromazine. Case report. Archives of disease in childhood. 1970;45(239):139-40.
38. Kullenberg F, Degerstedt O, Calitz C, Pavlovic N, Balgoma D, Grasjo J, Sjogren E, Hedeland M, Heindryckx F, Lennernas H. In Vitro Cell Toxicity and Intracellular Uptake of Doxorubicin Exposed as a Solution or Liposomes: Implications for Treatment of Hepatocellular Carcinoma. Cells. 2021;10(7).
39. Dinter J, Friedrich RP, Yang H, Pilarsky C, Mangge H, Pottler M, Janko C, Alexiou C, Lyer S. Mitoxantrone and Mitoxantrone-Loaded Iron Oxide Nanoparticles Induce Cell Death in Human Pancreatic Ductal Adenocarcinoma Cell Spheroids. Materials. 2023;16(7).

40. Chavan S, Parekh H, Chitnis M. Antineoplastic activity of mitoxantrone and its biological interactions in parental and multidrug resistant subline of P388 murine leukemia cells. Neoplasma. 1992;39(1):49-57.
41. Nagheh Z, Irani S, Mirfakhraie R, Dinarvand R. SN38-PEG-PLGA-verapamil nanoparticles inhibit proliferation and downregulate drug transporter ABCG2 gene expression in colorectal cancer cells. Progress in biomaterials. 2017;6(4):137-45.
42. Chen M, Li W, Zhang X, Dong Y, Hua Y, Zhang H, Gao J, Zhao L, Li Y, Zheng A. In vitro and in vivo evaluation of SN-38 nanocrystals with different particle sizes. International journal of nanomedicine. 2017;12:5487-500.
43. Penketh PG, Baumann RP, Ishiguro K, Shyam K, Seow HA, Sartorelli AC. Lethality to leukemia cell lines of DNA interstrand cross-links generated by Cloretazine derived alkylating species. Leukemia research. 2008;32(10):1546-53.
44. Williams CS, Watson AJ, Sheng H, Helou R, Shao J, DuBois RN. Celecoxib prevents tumor growth in vivo without toxicity to normal gut: lack of correlation between in vitro and in vivo models. Cancer research. 2000;60(21):6045-51.
45. Chen L, He Y, Huang H, Liao H, Wei W. Selective COX-2 inhibitor celecoxib combined with EGFR-TKI ZD1839 on non-small cell lung cancer cell lines: in vitro toxicity and mechanism study. Medical oncology. 2008;25(2):161-71.
46. Stuhlmeier KM. Effects of quinacrine on endothelial cell morphology and transcription factor-DNA interactions. Biochimica et biophysica acta. 2001;1524(1):57-65.
47. Changchien JJ, Chen YJ, Huang CH, Cheng TL, Lin SR, Chang LS. Quinacrine induces apoptosis in human leukemia K562 cells via p38 MAPK-elicited BCL2 down-regulation and suppression of ERK/c-Jun-mediated BCL2L1 expression. Toxicology and applied pharmacology. 2015;284(1):33-41.
48. Hyeon JW, Kim SY, Lee SM, Lee J, An SS, Lee MK, Lee YS. Anti-Prion Screening for Acridine, Dextran, and Tannic Acid using Real Time-Quaking Induced Conversion: A Comparison with PrPSc-Infected Cell Screening. PloS one. 2017;12(1):e0170266.
49. Buccarelli M, Marconi M, Pacioni S, De Pascalis I, D'Alessandris QG, Martini M, Ascione B, Malorni W, Larocca LM, Pallini R, Ricci-Vitiani L, Matarrese P. Inhibition of autophagy increases susceptibility of glioblastoma stem cells to temozolomide by igniting ferroptosis. Cell death & disease. 2018;9(8):841.
50. al-Rafaie FN, Wilkes S, Wonke B, Hoffbrand AV. The effect of deferiprone (L1) and desferrioxamine on myelopoiesis using a liquid culture system. British journal of haematology. 1994;87(1):196-8.
51. Fiorillo M, Toth F, Brindisi M, Sotgia F, Lisanti MP. Deferiprone (DFP) Targets Cancer Stem Cell (CSC) Propagation by Inhibiting Mitochondrial Metabolism and Inducing ROS Production. Cells. 2020;9(6).
52. Cserepes M, Turk D, Toth S, Pape VFS, Gaal A, Gera M, Szabo JE, Kucsma N, Varady G, Vertessy BG, Streli C, Szabo PT, Tovari J, Szoboszlai N, Szakacs G. Unshielding Multidrug Resistant Cancer through Selective Iron Depletion of P-Glycoprotein-Expressing Cells. Cancer research. 2020;80(4):663-74.
53. de Faria PA, Bettanin F, Cunha RL, Paredes-Gamero EJ, Homem-de-Mello P, Nantes IL, Rodrigues T. Cytotoxicity of phenothiazine derivatives associated with mitochondrial dysfunction: a structure-activity investigation. Toxicology. 2015;330:44-54.
54. Moraes VWR, Santos VM, Suarez ER, Ferraz LS, Lopes RM, Mognol GP, Campeiro JD, Machado-Neto JA, Nascimento FD, Hayashi MAF, Tersariol ILS, Newmeyer DD, Rodrigues T. Targeting Ca(2+) and Mitochondrial Homeostasis by Antipsychotic Thioridazine in Leukemia Cells. Life. 2022;12(10).
